# Redifferentiation of aged human articular chondrocytes by combining bone morphogenetic protein-2 and melanoma inhibitory activity protein in 3D-culture

**DOI:** 10.1371/journal.pone.0179729

**Published:** 2017-07-13

**Authors:** Stephan Payr, Brigitte Tichy, Clemens Atteneder, Marc Michel, Thomas Tiefenboeck, Nikolaus Lang, Sylvia Nuernberger, Stefan Hajdu, Elizabeth Rosado-Balmayor, Stefan Marlovits, Christian Albrecht

**Affiliations:** 1 Department of Trauma Surgery, General Hospital, Medical University of Vienna, Austria; 2 Austrian Cluster for Tissue Regeneration, Vienna, Austria; 3 Department of Experimental Trauma Surgery, Klinikum rechts der Isar, Technical University Munich, Germany; University of Maryland School of Medicine, UNITED STATES

## Abstract

Melanoma inhibitory activity (MIA) affects the differentiation to hyaline cartilage and can inhibit the osteogenic potential of bone morphogenetic protein (BMP)-2 in mesenchymal stem cells (MSC). The aim of this study was to investigate if MIA also inhibits the osteogenic potential of BMP-2 in human articular chondrocytes during redifferentiation, which may lead to a higher grade of differentiation without calcification. HAC of four female patients (mean age: 73.75 ±6.98) were seeded into 3D culture for 28 days; after adding the recombinant proteins, four groups were formed (Control, BMP-2, MIA, BMP-2+MIA). Samples were analysed for gene expression, glycosaminoglycan (GAG) content and histology on day 0, 14 and 28. Collagen type 2 (*COL2A1*) was significantly increased in the BMP-2 containing groups on day 28; BMP-2 (100-fold, p = 0.001), BMP-2+MIA (65-fold, p = 0.009) and similar to the level of native cartilage. Higher aggrecan (*Agg*) levels were present in the BMP-2 (3-fold, p = 0.007) and BMP-2+MIA (4-fold, p = 0.002) group after 14 days and in the BMP-2 (9-fold, p = 0.001) group after 28 days. Collagen type 10 (*COL10A1*) was increased in the BMP-2 containing groups (6-fold, p = 0.006) but these levels were significantly below native cartilage. Alkaline phosphatase (*ALP*), collagen type 1 (*COL1A1*) and the glycosaminoglycan (GAG) content did not reveal any relevant differences between groups. BMP-2 is a potent inducer for differentiation of HAC. A significant enhancement of this effect in combination with MIA could not be observed. Furthermore no significant reduction of osteogenic markers during re-differentiation of chondrocytes was present combining BMP-2 and MIA.

## Introduction

Chondral lesions caused by trauma, biomechanical misloading or degeneration show a high incidence and are still challenging in orthopaedic and trauma surgery [1–5]. Deep osteochondral defects can heal with inferior fibrocartilage via immigration of mesenchymal stem cells [6]. New approaches try to enhance full regeneration of the chondral defect with hyaline cartilage and therefore to restore full articular function. The matrix-associated autologous chondrocyte transplantation (MACT) uses the chondrogenic potential of autologously transplanted chondrocytes for producing hyaline-like cartilage.

Highly differentiated chondrocytes are needed for this method to achieve hyaline-like cartilage. Studies indicate that a high grade of differentiation at the time of transplantation is positively connected to a better clinical outcome [7]. However, the limitation of this tissue engineering method is the dedifferentiation of chondrocytes during proliferation in monolayer culture [8–13]. Monolayer cultivation is necessary for proliferation of chondrocytes, but this is not comparable to physiological conditions.

A promising method to achieve a native phenotype in dedifferentiated chondrocytes seems to be the introduction of differentiation factors in order to induce (re-)differentiation. A supportive regulator for chondrogenic differentiation is bone morphogenetic protein-2 (BMP-2) [14]. However, BMP-2 is also a potent inducer for bone healing. Therefore, in chondrogenic differentiation, BMP-2 may lead to hypertrophy and calcification of mesenchymal stem cells differentiating into chondrocytes [15–17]. A possibility to ease this challenge may be the melanoma inhibiting activity protein (MIA), also referred to as cartilage-derived retinoic acid sensitive protein (CD-RAP). This is a small soluble protein originally isolated from malignant melanoma cells and later from cartilage. MIA is mainly expressed in embryonic and adult cartilage under physiological conditions. Pathologically, MIA is expressed in chondrosarcoma, melanoma, breast cancer and elevated serum levels can be found in rheumatoid arthritis and osteoarthritis. The physiological function of MIA in cartilage is still not fully understood. However, recent studies revealed that MIA can modulate BMP-2 induced differentiation into a chondrogenic direction in human mesenchymal stem cells (hMSC) [18]. MIA itself is not capable of inducing differentiation of hMSC, but influences the action of BMP-2 by up-regulating chondrogenic markers and down-regulating osteogenic markers suggesting an inhibition of the osteogenic potential of BMP-2 [19]. The main focus of all these studies was the effect of MIA and BMP-2 on hMSC. Therefore, we were interested in investigating this observed effect of BMP-2 and MIA in primary human articular chondrocytes.

The aim of this study was to investigate if MIA also modulates the effect of BMP-2 in human articular chondrocytes during redifferentiation, which may lead to a higher grade of differentiation without calcification. This could further lead to a better clinical outcome for patients treated with MACT.

## Methods and methods

### Isolation and cultivation of human articular chondrocytes

Human articular cartilage samples were collected from the femoral heads of four female patients (mean age: 73.75 ±6.98) with no history of joint disease, who were scheduled to undergo joint replacement due to femoral neck fracture (approved by the Ethics Board of the University of Vienna; code: 1949/2014 S1 Fig; the study was conducted according to the Declaration of Helsinki in its latest amendment; all four patients signed patient’s information and informed consent which was prior approved by the Ethics Board of the University of Vienna). Cartilage with no macroscopic signs of osteoarthritis was dissected from the bone immediately after removal of the femoral heads. Cartilage pieces were put into an antibiotic solution consisting of phosphate-buffered saline (PBS) (PAA), amphotericin B (PAA) and gentamycin (Gibco). The cartilage specimens were digested with 1mg/ml hyaluronidase (Sigma-Aldrich), 1 mg/ml Pronase (Roche) and 200 U/ml collagenase in Dulbecco’s Modified Eagle Medium (DMEM) for 1 day at 37°C. The obtained cell suspension was passed through a 40 μm nylon mesh cell strainer and centrifuged at 450 g for 10 min. The cell pellet was resuspended in DMEM with high glucose (Gibco), 10% fetal calf serum (FCS), 2 μg/ml amphotericin B (Lonza), 100 μg/ml gentamycin (Gibco), 50 μg/ml L-ascorbic acid 2-phoshate (Sigma-Aldrich), 10 mM HEPES (Gibco), 5 μg/ml insulin (Sigma) and L-glutamine (Gibco). The cells were isolated and cultured in monolayer according to our standard operation procedure, as previously described [8]. From the same cartilage, which was used for cell isolation, a fraction was used as native cartilage control.

The chondrocytes were proliferated in monolayer culture until passage P2 with a population doubling (PD) of 3.2 ±0.6 (PD was calculated according to the formular PD = log(N2/N1)/log2 where N1 is the number of seeded cells and N2 number of cells after passaging) and then transferred to 3D culture in alginate. For the preparation of alginate beads, 1.25% sodium alginate (Sigma, Fluka) in 0.15 M NaCl (0.9%) was used. The cells were resuspended in the alginate solution at a density of 4x10^6^ cells/ml. The cell suspension was dropped into a 102 mM CaCl2 solution using a 22-gauge needle and incubated at 37°C for 20 min, washed three times and put into culture medium as described above. After two days, alginate beads were randomly divided into four groups—26 beads per group per donor—(Group 1: Control (CO), Group 2: BMP-2, Group 3: MIA, Group 4: BMP-2 + MIA) and the recombinant proteins were added for the first time. Concentrations for the recombinant proteins were as follows: 100 ng/ml BMP-2 (Sigma-Aldrich) and 500 ng/ml MIA (Sigma-Aldrich). Changing the medium solution and adding the recombinant proteins was performed every second day. Native cartilage (referred in diagrams only as native) and dedifferentiated cells prior to 3D cultivation as a second control group (referred in diagrams only as cells) were used as additional control groups. The times of observation were: prior to adding the recombinant proteins (CO d0), after 14 days (d14) and after 28 days in alginate culture (d28). Beads were used as a whole for RNA isolation following gene expression analysis with real-time PCR, investigation of glycosaminoglycan (GAG) content and histological evaluation.

### RNA extraction and purification

Isolation of total RNA was performed with the RNeasy Plus Mini Kit (Qiagen) according to the manufacturer’s instructions. For total RNA extraction from the alginate beads, beads were transferred to a 1.5 ml tube with a solution provided by the kit and disrupted by a sterile pestle until no signs of beads were remaining. For total RNA extraction from native cartilage, slices of human articular cartilage were frozen in liquid nitrogen and ground using a mortar and pestle. Further steps were performed using the RNeasy Plus Mini Kit according to the manufacturer’s instructions (Qiagen, Germany).

### cDNA synthesis

Total RNA (0.1–1 μg) was diluted with nuclease-free water to a volume of 15 μl. Thereafter, 4 μl iScript reaction mix, as well as 1 μl iScript reverse transcriptase were added (Bio-Rad Laboratories, Hercules, California). The reaction mix was incubated for 5 min at 25°C, for 30 min at 40°C and for 5 min at 85°C.

**Real-time polymerase chain reaction (PCR)** amplification was performed and monitored using an ABI PRISM 7500 fast real-time PCR System (Applied Biosystems). The master mix was based on the SensiMix Probe Kit (Quantace, London, United Kingdom). The thermal cycling conditions comprised the initial steps at 50°C for 2 min and at 95°C for 10 min. Amplification of the cDNA products was performed with 40 PCR cycles, consisting of a denaturation step at 95°C for 15 sec and an extension step at 60°C for 1 min. β-2-microglobulin (B2M) was chosen as the housekeeping gene, using the pre-developed Taq Man assay (Applied Biosystems). *COL1A1*, *COL2A1* and *Agg* primers were designed by the authors [20]. *COL10A1* and *ALP* primers were pre-developed Taq Man assay (Applied Biosystems). All cDNA samples (2.4 μl of cDNA in a total volume of 20 μl) were analysed in triplicates. Relative quantification of gene expression was performed using the comparative ΔΔCt method. Native cartilage was used as a calibrator.

**Glycosaminoglycan and picogreen assay** were performed in each sample with the Blyscan GAG Elisa Kit (Biocolor, Edition 2011, UK). To determine the amount of DNA in each sample, the Quant-iT PicoGreen dsDNA Reagent and Kits (Invitrogen) were used. Briefly, alginate was dissolved with papain extraction reagent according to the Biocolor handbook. Two alginate beads were incubated with 1 ml papain extraction reagent in 1.5 ml microcentrifuge tubes. Overnight, the tubes were put into a water bath at 65°C. Samples for Picogreen testing were taken. Tubes were then centrifuged at 10 000 g for 10 minutes. The supernatant was used in the Blyscan GAG Assay. Samples were measured in duplicates at 656 nm (Victor 2, HVD Life Science). The PicoGreen-treated samples were measured in duplicates at 480/529 nm (Victor 3, Perkin Elmer). The ratio of GAG and DNA content of each sample was calculated and expressed as μg GAG per μg DNA.

### Histology

For histological examinations standard protocols were used. In brief, cells were treated with 0.1 M sodium cacodylate buffer with 100 mM barium chloride (BaCl_2_) for 30 min. Subsequently, beads were fixed with 7.5% formaldehyde for 60 min at room temperature and later kept in 0.1 M cacodylate buffer with 50 mM BaCl_2_. Deparaffinized sections were stained with alcian blue.

### Statistical analysis

Each experiment was independently performed four times with chondrocytes from four different donors. For GAG/DNA assay and real-time PCR, all samples were analyzed in triplicates. Gene expression data were reported as the mean ± standard deviation of the real-time PCR analyses and expressed in log scales. For statistical analysis, ΔΔCt values were used. Statistical analysis was performed using SPSS software version 20.0 (SPSS Inc, Chicago, Illinois).

General linear model repeated measures procedure was used for comparison of samples at different time points. Differences between the treated groups were analysed by paired t-tests.

Alpha error was set at 0.05. Bonferroni—Holm correction was performed to prevent alpha error accumulation.

## Results

### Gene expression

#### Collagen type 2

**Fig 1:**
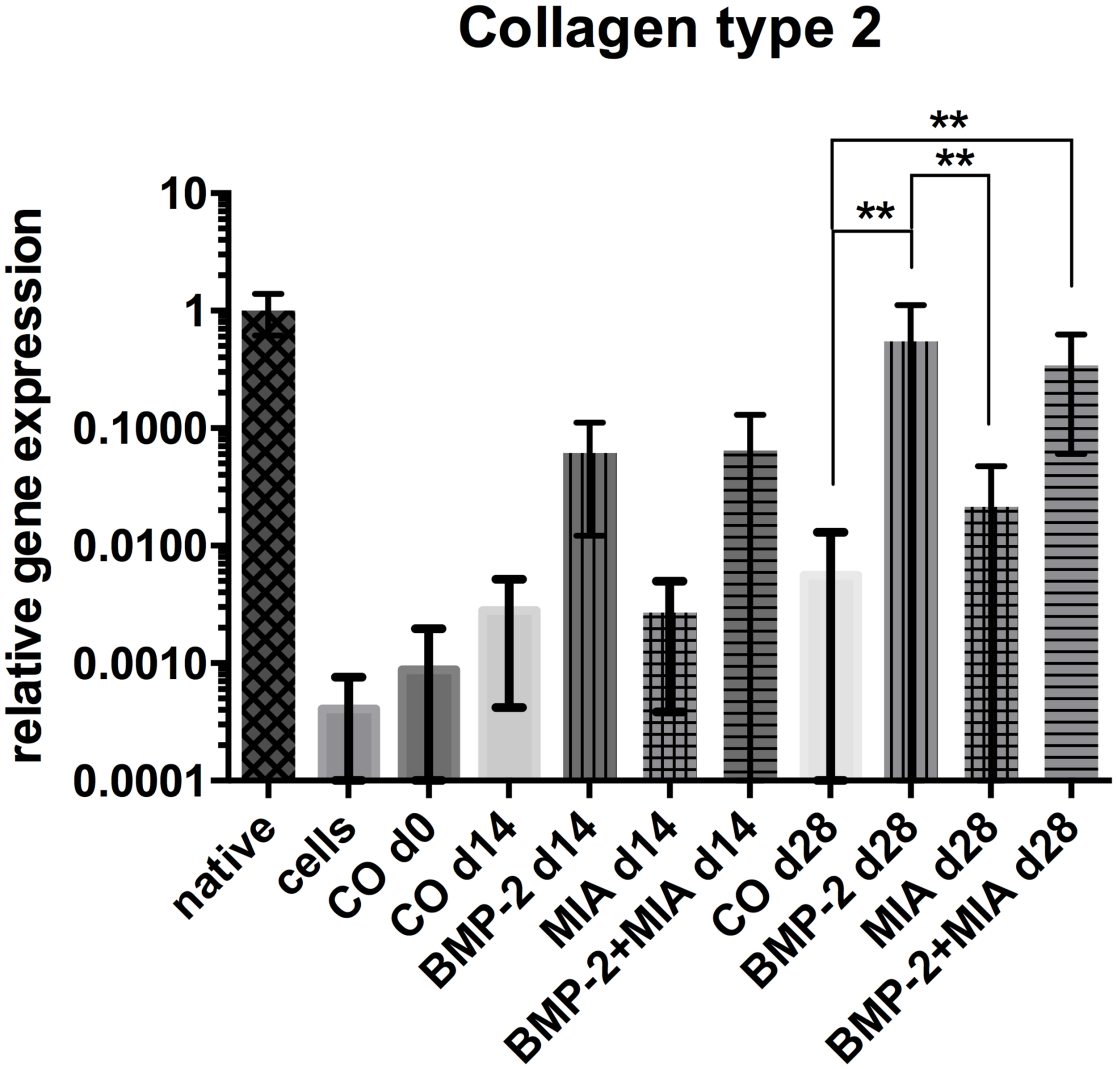
Relative gene expression of *COL2A1*. Relative gene expression of *COL2A1* normalised to native cartilage in log scale. Comparison of native cartilage (native), dedifferentiated cells—prior to 3D cultivation (cells), control on day 0 (CO d0), control and treated groups on day 14 and 28. * (p<0.05), ** (p<0.01).

After 2 weeks, a tendency of increased expression of *COL2A1* in both groups containing BMP-2 was present (BMP-2 d14, BMP-2+MIA d14) (Fig 1) S2 Fig. After 4 weeks, we observed a >100-fold increase of the *COL2A1*expression in the BMP-2 only treated beads (BMP-2 d28) versus the control (CO d28) (p<0.0001). Comparing BMP-2 + MIA on day 28 (BMP-2+MIA d28) with the control (CO d28), the combination showed an over 65-fold increase of *COL2A1* (p = 0.002). The BMP-2 only group (BMP-2 d28) had a 25-fold higher level of *COL2A1* than the beads with MIA (MIA d28) on day 28 (p = 0.009).

The chondrocytes treated with BMP-2 for 28 days (BMP-2 d28) showed an almost similar *COL2A1* expression level as the native cartilage (native).

The control group at d0 (CO d0) did not differ significantly from the control at day 14 (CO d14) and 28 (CO d28), but a slightly increasing tendency was observed during the whole observation period.

#### Aggrecan

**Fig 2:**
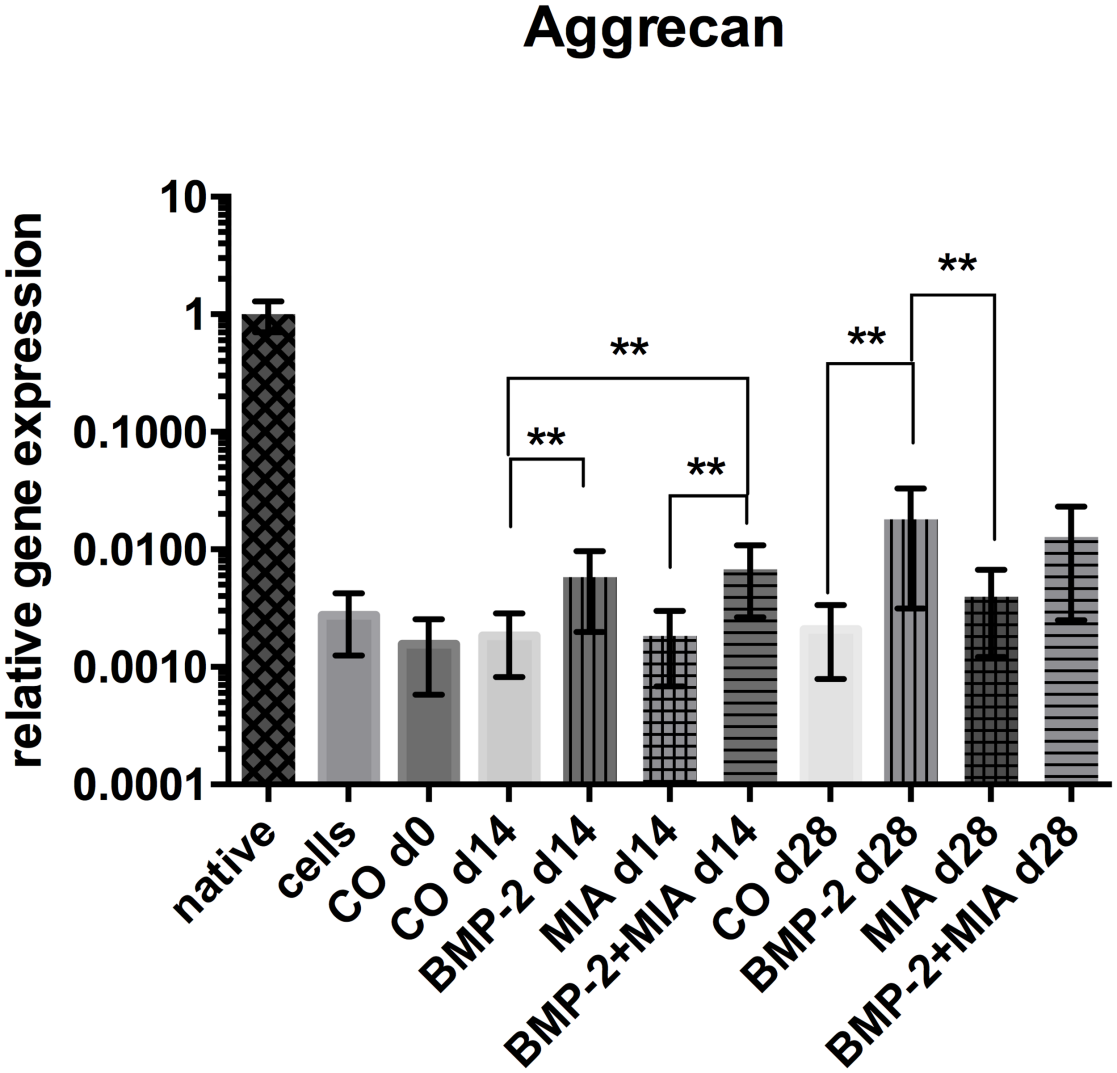
Relative gene expression of *Agg*. Relative gene expression of *Agg* normalised to native cartilage in log scale. Comparison of native cartilage (native), dedifferentiated cells—prior to 3D cultivation (cells), control on day 0 (CO d0), control and treated groups on day 14 and 28. * (p<0.05), ** (p<0.01).

On day 14, a significant up-regulation of *Agg* levels was detected in the groups containing BMP-2 (BMP-2 d14, BMP-2+MIA d14) when compared with the control (CO d14);

BMP-2+MIA presented an over 4-fold increase (p = 0.002), BMP-2 a 3-fold increase (p = 0.007) (Fig 2). MIA alone (MIA d14) however produced lower *Agg* levels (3.6-fold) than the combination of BMP-2+MIA on day 14 (p = 0.008).

After four weeks, significant rises of *Agg* still appear in the BMP-2 group (BMP-2 d28) when compared with CO d28 (9-fold; p = 0.001) and the MIA (MIA d28) (>4-fold; p = 0.002) group. Within the last 2 weeks, the *Agg* levels doubled in the MIA group. All groups expressed less *Agg* than the native control. The control groups only showed a mild increase during the course of the study.

#### Collagen type 1

**Fig 3:**
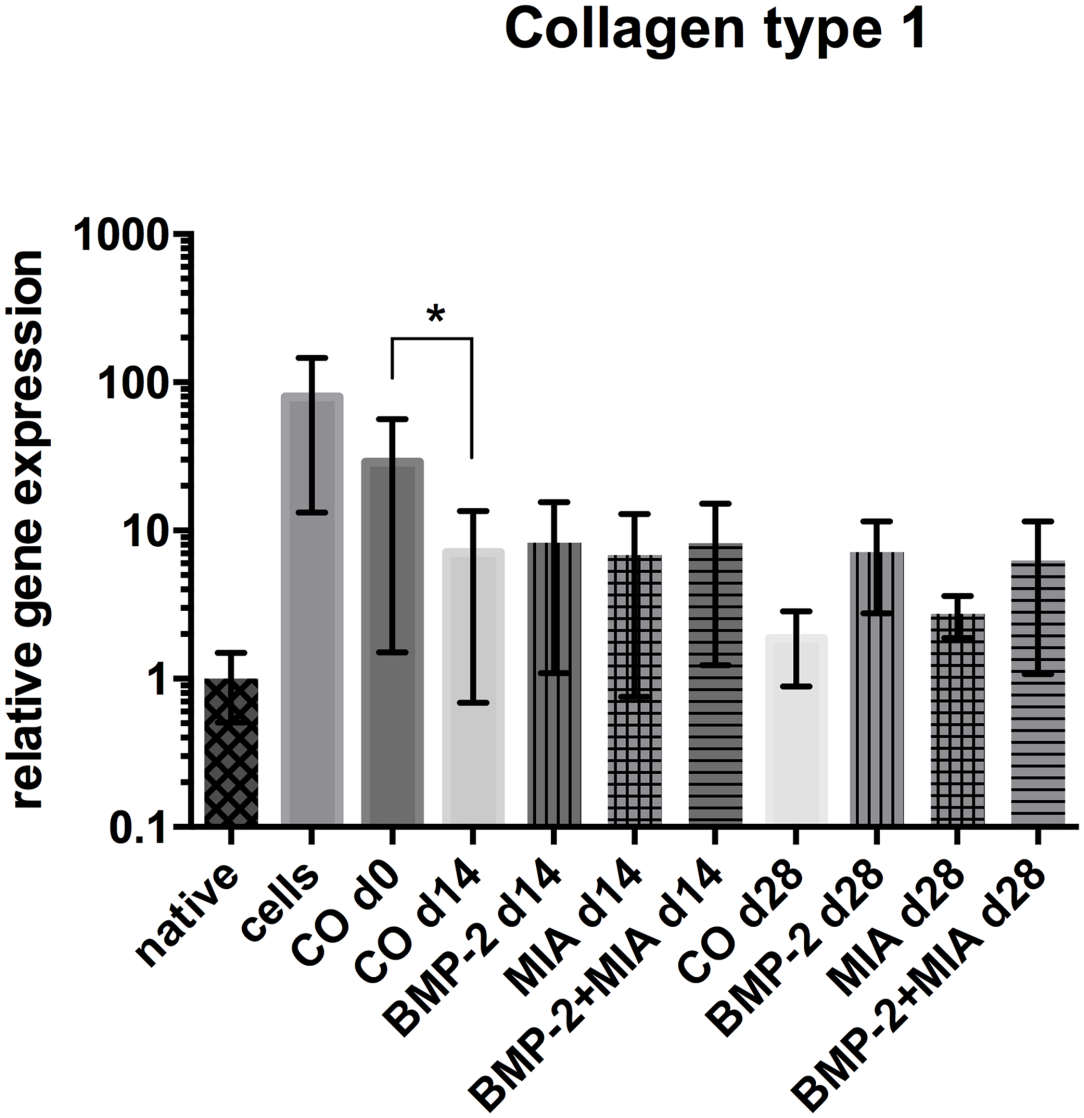
Relative gene expression of *COL1A1*. Relative gene expression of *COL1A1* normalised to native cartilage in log scale. Comparison of native cartilage (native), dedifferentiated cells—prior to 3D cultivation (cells), control on day 0 (CO d0), control and treated groups on day 14 and 28. * (p<0.05), ** (p<0.01).

The expression levels of *COL1A1* appeared similar between all treated groups compared to the control group (CO d14) on day 14 (Fig 3). In the following two weeks, the *COL1A1* expression levels in the BMP-2 containing groups did not change significantly but decreased levels in the combination group could be seen. The MIA group (MIA d28) exhibited the lowest *COL1A1* levels of the treated groups on day 28. When comparing the control groups (CO d0, CO d14 and CO d28) a significant reduction of *COL1A1* between d0 and on day 14 (p = 0.016) was present and also showed this decreasing tendency after 28 days.

#### Collagen type 10

**Fig 4:**
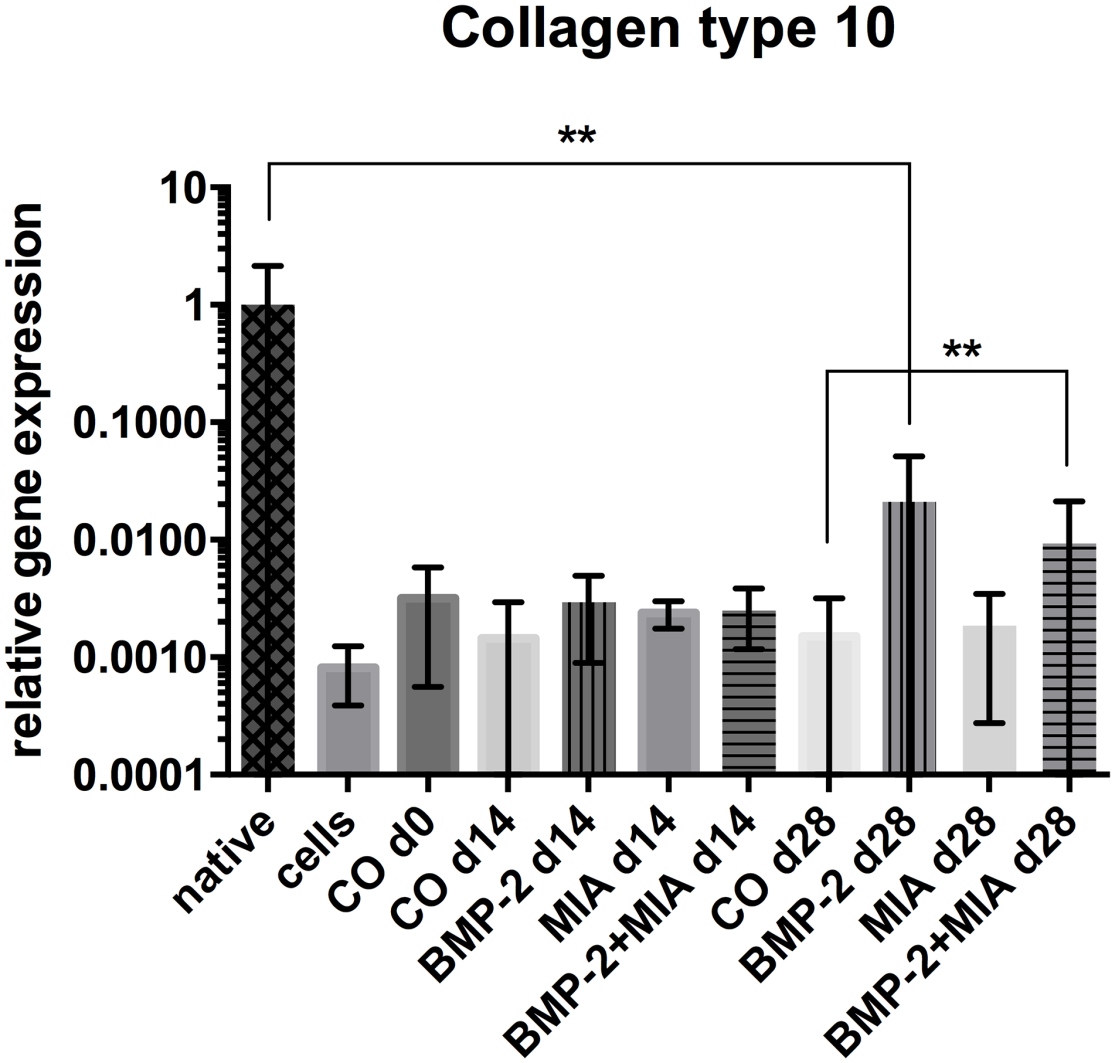
Relative gene expression of *COL10A1*. Relative gene expression of *COL10A1* normalised to native cartilage in log scale. Comparison of native cartilage (native), dedifferentiated cells—prior to 3D cultivation (cells), control on day 0 (CO d0), control and treated groups on day 14 and 28. * (p<0.05), ** (p<0.01).

After 14 days, only the BMP-2 group (BMP-2 d14) had slightly elevated *COL10A1* levels compared to the control group (CO d14) (Fig 4). The levels in both of the MIA-containing groups (MIA d14, BMP-2+MIA d14) were similarly low and slightly beneath the control group (CO d14). *COL10A1*expression analysis revealed a significant increase between the control (CO d28) and the BMP-2+MIA group (p = 0.006) on day 28. The BMP-2 group (BMP-2 d28) showed by trend the highest levels of *COL10A1*, but without statistical significance. A reduction of over one third from the highest levels of the BMP-2 group (BMP-2 d28) to the levels of the combination group (BMP-2+MIA d28) could be observed, but this was not statistically significant. The control groups at different time points indicated a decreasing tendency after 4 weeks. Nevertheless, a high reduction (over 47-fold) of *COL10A1* expression is noticeable when comparing the native cartilage (native) to even the highest levels in the BMP-2 group on day 28 (p = 0.004).

#### Alkaline phosphatase

**Fig 5:**
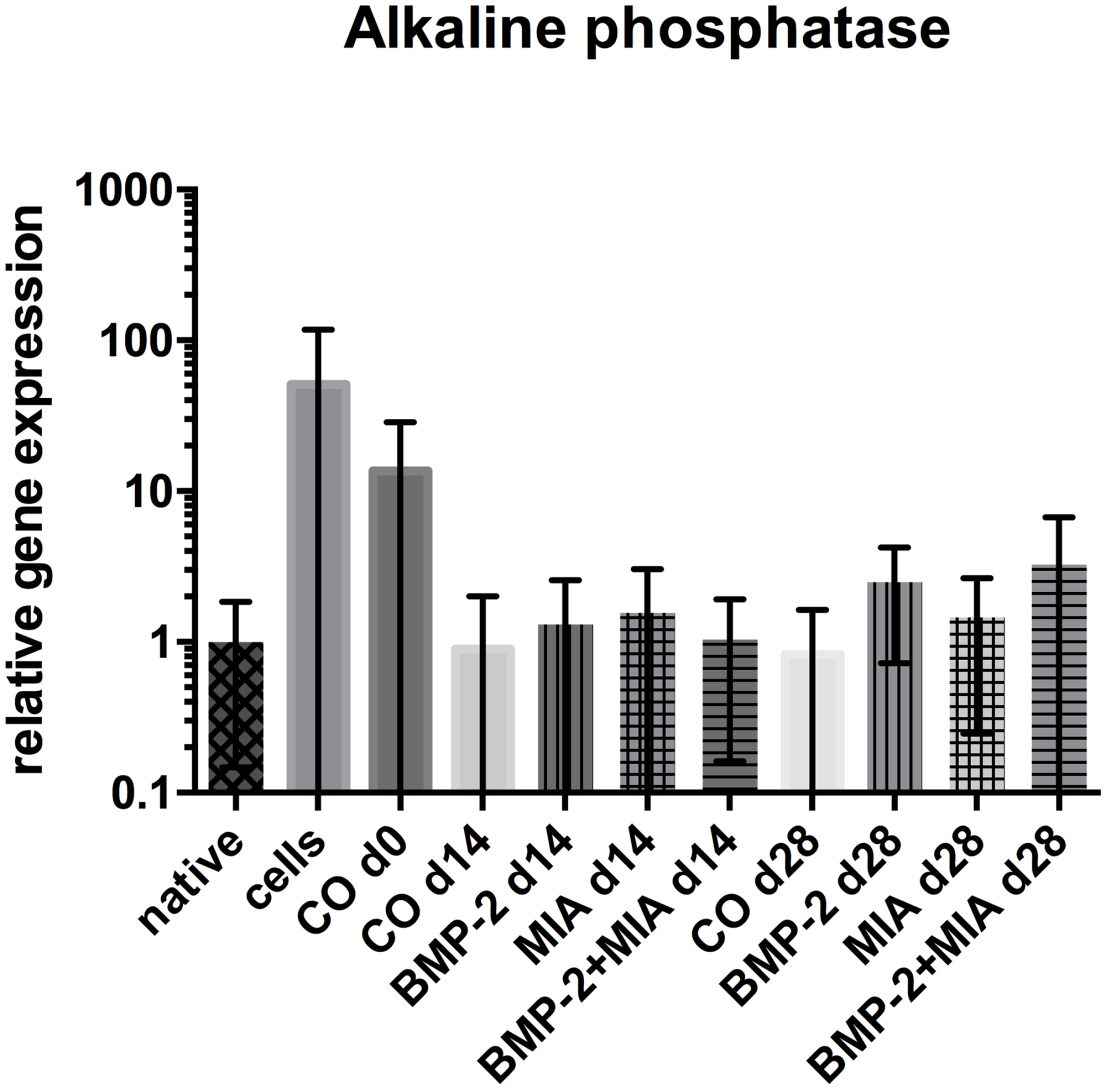
Relative gene expression of *ALP*. Relative gene expression of *ALP* normalised to native cartilage in log scale. Comparison of native cartilage (native), dedifferentiated cells—prior to 3D cultivation (cells), control on day 0 (CO d0), control and treated groups on day 14 and 28. * (p<0.05), ** (p<0.01).

There were no statistically significant results found in the expression of *ALP* between the groups at any time point (Fig 5). After 28 days, the groups containing BMP-2 (BMP-2 d28, BMP-2+MIA d28) tended to higher levels of *ALP* than the other groups and the native cartilage. The expression pattern of *ALP* is similar to *COL1A1*. Dedifferentiated monolayer cells and cells at the beginning of 3D culture (CO d0) showed highest expression of *ALP* and *COL1A1*.

#### Glycosaminoglycan content

**Fig 6:**
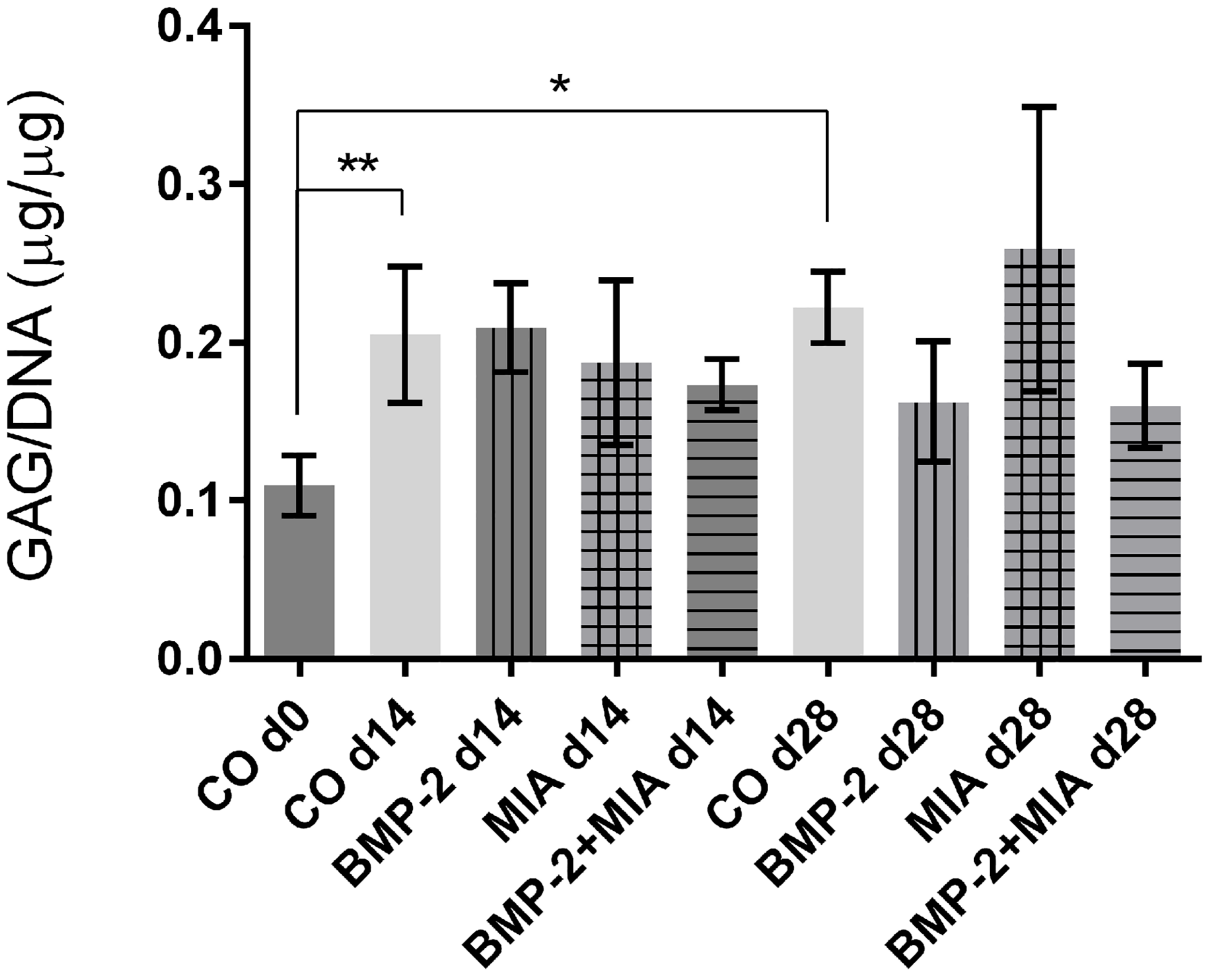
Glycosaminoglycan content in relation to DNA content. Comparison of control on day 0 (CO d0), control and treated groups on day 14 and 28. * (p<0.05), ** (p<0.01).

Between the groups, no significant differences were seen concerning the GAG content in relation to the number of cells (Fig 6). An increase was found only when comparing CO d0 with CO d14 (p = 0.003) and CO d0 with the CO d28 (p = 0.014).

#### Histology

**Fig 7:**
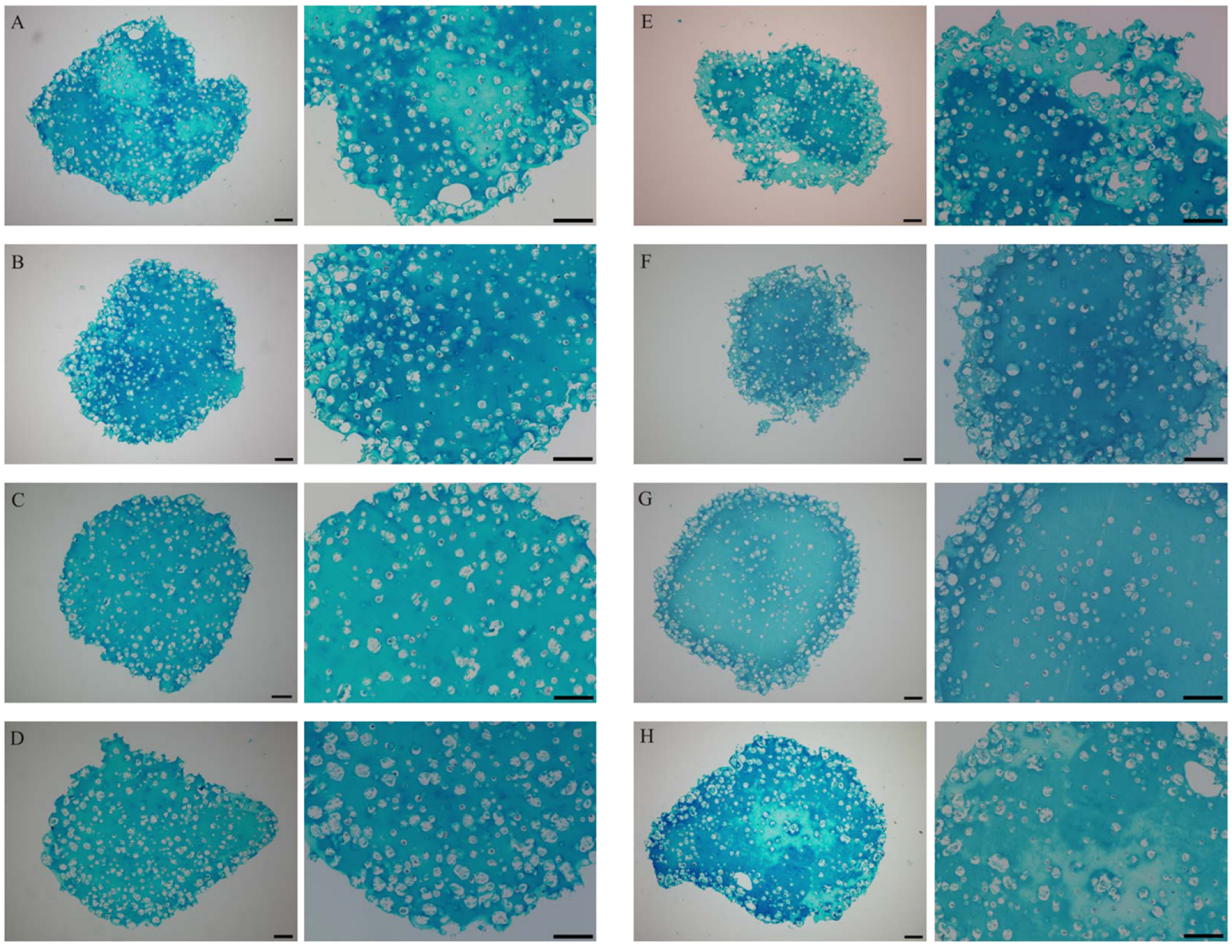
Alcian blue staining of alginate beads in 10 and 20 times magnification. Scale in each slide corresponds to 100μm. (A) Control on day 14; (B) BMP-2 on day 14 (C) MIA on day 14; (D) BMP-2+MIA on day 14; (E) Control on day 28; (F) BMP-2 on day 28; (G) MIA on day 28; (H) BMP-2+MIA on day 28.

The Alcian blue staining (Fig 7) indicating the GAG content, showed a darker blue on the outside margin of the control bead on day 14 (A). BMP-2 on day 14 (B) seemed to be more homogenous and more intense blue at a similar cellular content and arrangement compared to CO14. MIA on day 14 (C) was lighter but no signs of cell loss or dedifferentiation were visible. The combination of BMP-2 and MIA on day 14 (D) seemed to produce 3 kinds of blue intensities throughout the sample. It appeared to be lighter with single dark spots in the centre and darker areas on the outside margin.

Interestingly, on day 28, the control (E) was the opposite of day 14 being darker in the centre and lighter on the outside margins.

BMP-2 on day 28 (F) was again homogenous and dark blue. MIA on day 28 (G) appeared to be lighter again and cells seemed to be rather situated in the centre.

The combination of BMP-2 and MIA on day 28 (H) was intense blue and in contrast to CO28, the bead was rather intense blue on the outside margins. In this bead, cells seemed to be situated near the outside margin.

## Discussion

In this study, we observed a distinct increase of *COL2A1* expression in human articular chondrocytes when stimulated by BMP-2, indicating an enhanced chondrogenic differentiation. An over 100-fold expression compared to the control group seems to be consistent with the literature. Gründer et al. even report a 256-fold rise of *COL2A1* in HAC using BMP-2 in 3D culture. The same author states that BMP-2 had no effect on HAC in monolayer cultivation. Despite different settings, an increased collagen type II expression by BMP-2 induction is mentioned in the literature—independently in human or animal mesenchymal stem cells and at different dosages of BMP-2 for different time periods [21–25]. The 4-fold higher levels of *Agg* in the BMP-2-containing groups after 14 days are reported in current literature as well [21–23]. In this study, a 4-fold increase after 14 days was present and even 9-fold higher levels of *Agg* were observed after four weeks of BMP-2 stimulation strengthening the enhanced chondrogenic differentiation. This observation could also be shown in the histological sections. The alcian blue staining was more intense and homogenous in the BMP-2 groups. Interestingly, in the combination group on day 28, the outside margins were darker than the centre and cells seemed to have moved towards the outside margins of the bead. This might be an indication that the growth factors are active leading to increased metabolism and consumed nutrients so that the cells were forced to move towards the outside margin.

Hypertrophy and calcification of cells after induction with BMP-2 are issues postulated in the literature [16, 26]. These changes are characterised by an increased expression of common hypertrophic and osteogenic markers such as COL10 and ALP in chondrocytes and runt-related transcription factor 2 (Runx2), osteocalcin (OC), osteopontin (OP), ALP and osteoprotegerin (OPG) in MSCs [27–30]. The *COL10A1* expression in this study was increased in the combination group and in the BMP-2 only group in comparison to the control group after the whole period of 28 days.

This result is consistent with current literature. Murphy et al. seeded passaged HACs/hMSCs in an aggregate culture for only 7 days stimulating with 100 ng/ml BMP-2 and also found increased levels of *COL10A1* in comparison to an *in vitro* control group [31]. A further study with HACs after passage 2 in monolayer revealed elevated *COL10A1* levels compared to an *in vitro* control group when stimulated with BMP-2 [32]. In contrast to our study, no comparison to original native cartilage tissue is present in both of these papers. In our study, the distinct higher levels of *COL10A1* in the native cartilage compared to the highest levels of the BMP-2 only group noticeable are, indicating that the *COL10A1* levels and hypertrophy of cells are relatively negligible. As an osteogenic marker, we focused on *ALP*, but the gene expression did not identify significant differences between the groups. The expression pattern of dedifferentiated cells and the group at d0 are similar to those of *COL1A1*. The expression pattern of *ALP* in the treated groups is similar to the ones of *COL1A1* and *COL10A1* and treated groups show a minimal rising tendency compared to *ALP* levels in native cartilage. Data indicate that the issue of hypertrophy and expression of osteogenic markers after BMP-2 stimulation might be more relevant in MSCs than in HACs. In this study performed in HACs, we could not detect such a significant reduction of hypertrophic and osteogenic markers as authors testing the combination of BMP-2 and MIA in MSCs [18] [19].

MIA is a protein, expressed in developing and mature cartilage. Despite adding recombinant MIA to a 3D cultivation system, *COL2A1* and *Agg* levels are only increased by trend either as a relic of monolayer cultivation or because of inefficient differentiation in alginate beads [20]. 3D cultivation in alginate beads is a common system in cartilage research and used in similar studies [19].

In this study, MIA did not significantly improve chondrogenesis by elevating *COL2A1* and *Agg* levels when added to HAC in 3D cultivation. This observation is underlined histologically by the lighter shades in the alcian blue staining in the MIA group on day 28.

In the literature, only one paper deals with treated HAC in alginate beads with MIA. Tscheudschilsuren et al. only investigated the GAG levels, which were found to be increased [19]. In this setting, it was not capable to reproduce this result despite the same cultivation system and concentration of MIA. Histologically, MIA at both time points appeared lighter in the Alcian blue staining compared to the BMP-2-containing groups indicating less GAG.

A noticeable difference that might explain this observation is the donor age difference, mean age of 46.6 years (one donor even only 20 years old) vs. a mean age of 73.75 years in our study. Due to younger age, there might be a higher GAG deposition itself or maybe HACs in this study could be not as sensitive to MIA due to senescence of human articular chondrocytes leading to lower post-expansion chondrogenic capacity [33, 34].

Schubert et al. treated dedifferentiated human primary chondrocytes with 100 ng/ml MIA for 30 min in monolayer cultivation. They observed an increase of *Agg* and a decrease of *osteocalcin* compared to untreated dedifferentiated HACs [18].

Tscheudschilsuren et al. investigated the effect of MIA on BMP-2 induced hMSCs in 3D cultivation [19]. It was shown that MIA enhanced the chondrogenic induction of BMP-2 on hMSCs and further inhibited the osteogenic potential. *COL2A1* was significantly increased in the combination group and the osteogenic markers were significantly reduced. The 3D cultivation was pellet culture for 21 days and MIA was administered at a concentration of 500 ng/ml and BMP-2 of 100 ng/ml. MIA alone did also have no effect on hMSCs.

In this conducted study, the aim was to improve redifferentiation of human articular chondrocytes in 3D culture by combining BMP-2 and MIA and to see whether redifferentiation can still be enhanced while inhibiting osteogenic markers. The highest levels of *COL2A1* and *Agg* were found in the BMP-2 groups after 28 days followed by the combination. These results indicate that MIA was not able to enhance the chondrogenic effect in this setting. MIA is postulated to have a chemotactic effect to recruit mesenchymal stem cells [19]. While this capacity may affect cartilage repair *in vivo* by increasing the number of cells with chondrogenic potential, this effect has no impact on the chondrogenic differentiation *in vitro*.

Furthermore, in this setting, combining BMP-2 and MIA did not significantly decrease the expression of hypertrophic and osteogenic markers. MIA barely inhibited the *COL10A1* expression of BMP-2. The fact that the highest levels of *COL10A1* in the BMP-2-containing groups are over 47-fold lower than the levels of native cartilage indicates that the issue of hypertrophy and calcification by BMP-2 is more likely linked to MSCs than HACs. We decided to work with only one concentration of MIA (500ng/ml), because this concentration was usually mentioned for MSC but also once for HAC in the literature. Different variations and application patterns seemed to produce inferior results [19]. Due to the fact that high dosages of MIA may inhibit the chemotaxis, the question arises if on the one hand this concentration may have some sort of inhibitory effect on HACs. On the other hand, this concentration of MIA might be too low to have a significantly positive effect on HACs or MIA is simply not capable of enhancing chondrogenesis of HACs. In order to further investigate this issue, different dosages of MIA would have been needed. Another limitation of this study may be the use of only one cultivation method, but alginate beads are commonly used for HAC and MSC in the literature [22, 35, 36].

The high standard deviation leading to extended error bars in the diagrams is noticeable in our results. This high standard deviation can be explained by high interindividual differences of gene expression between the donors. Due to the fact that rises of gene expression in the BMP-2 groups were seen in every donor, non-responders to BMP-2 can be excluded.

To date, this study has the longest observation period concerning this topic and samples where taken at 3 time points. Current papers report observation periods between 30 min and 21 days and samples were taken at only one time point [18, 19]. One unique aspect of this study is the evaluation and observance of native cartilage, cells and the control at day 0. Especially the native cartilage allows it to bring these results in relation to physiologic conditions. On the one hand, native cartilage emphasizes the results of *COL2A1* and on the other hand relativises the results of *COL10A1*, making even the highest levels of *COL10A1* negligible despite significant elevation in-vitro.

In summary, BMP-2 seems to be a potent and feasible differentiation factor for HACs, since stimulated osteogenic induction may not be relevant for HACs. MIA did neither enhance chondrogenic differentiation nor inhibit osteogenesis of HAC when combined with BMP-2.

## Supporting information

S1 Fig Vote-signed-at-20150303. Statement of the Ethics Board of the University of Vienna.
(PDF)

S2 Fig Minimal data set. Minimal data set for each gene and the GAG/DNA.
(PDF)
