# Supplementary material for: Redifferentiation of aged human articular chondrocytes by combining bone morphogenetic protein-2 and melanoma inhibitory activity protein in 3D-culture
Source: PLoS One. 2017 Jul 13;12(7):e0179729. doi: 10.1371/journal.pone.0179729 (PMC5509113; doi:10.1371/journal.pone.0179729)
Supplement: S1 Fig — Statement of the Ethics Board of the University of Vienna. (PDF) [file pone.0179729.s001.pdf]

ethik kommission
Medizinische Universität Wien

Borschkegasse 8b/6
A-1090 Wien, Austria

T +43 1 40 400 – 21470, 22440
F +43 1 40 400 – 16900
ethik-kom@meduniwien.ac.at
ethikkommission.meduniwien.ac.at

Votum:

EK Nr: 1949/2014
Projekttitel: Verbesserung der chondrogenen Differenzierung durch Modulation der Bone Morphogenetic Protein 2 (BMP-2) Wirkung mittels Melanoma Inhibitory Activity (MIA)
Antragsteller/in: Herr Dr. Stephan Payr
Institution: AKH WIien/Unfallchirurgie
Sponsor: MUW

Teilnehmende Prüfzentren:

Ethik-Kommission	Prüfzentrum	Prüfärztin/arzt
Ethikkommission der Medizinischen Universität Wien	Univ.-Klinik für Unfallchirurgie	Herr DDr. Christian Albrecht

Die Stellungnahme der Ethik-Kommission erfolgt aufgrund folgender eingereichter Unterlagen:

Dokument	Name	Version	Datum
Conflict of Interest	Conflict of interest	version 1	03.12.2014
Lebenslauf (CV)	CV Albrecht	Version 1	28.10.2014
Patienteninformation	patinfo ek BMP-2+MIA Version 2 23012015	Version 2	23.01.2015
	patinfo ek version 1	version 1	03.12.2014
Studienprotokoll (Prüfplan)	Verbesserung der chondrogenen Differenzierung durch Modulation der Bone Morphogenetic Protein 2	Version 1	03.12.2014

Die Kommission fasst folgenden Beschluss (mit X markiert):

☒	Es besteht kein Einwand gegen die Durchführung der Studie.

Ergänzende Kommentare der Sitzung am 13.01.2015:
Zum Antrag:
Punkt 1.1: Der englische Studientitel sollte vermutlich "Improvement of chondrogenic differentiation by BMP-2 and MIA combination" heißen.
Die Schreibfehler, insbesondere in der Kurzfassung, sind zu korrigieren.

Zur Patienteninformation:
Punkt 2: "OP" ist auszuschreiben.
Punkt 9: Es ist zu ergänzen, dass auch keine Vergütung vorgesehen ist.

Punkt 10: Die Telefonnummer des AKH Wien ist zu aktualisieren (an die Klappennummer ist eine Null anzuhängen). Ist unter der angeführten Telefonnummer tatsächlich ständige Erreichbarkeit gegeben? Wenn dies nicht der Fall ist, so ist das Wort "ständig" zu streichen.
Punkt 11: Im Text ist der falsche Studientitel angegeben, dies ist zu korrigieren. "OP" ist auszuschreiben.

Zur Versicherung: nicht erforderlich

Andere:
Das unterschriebene Antragsformular ist nachzureichen.

Die Ethik-Kommission ersucht die Antragsteller, bei der Wiedervorlage von geänderten Unterlagen ein Exemplar mit hervorgehobenen Änderungen beizulegen.

Ergänzende Kommentare:
Nachtrag vom 3. März 2015:
Die Antragsteller legen am 23.01.2015 überarbeitete Unterlagen vor, die von der Ethik-Kommission akzeptiert werden. Das unterschriebene Antragsformular wurde nachgereicht.

Die aktuelle Mitgliederliste der Ethik-Kommission ist unter der Adresse http://ethikkommission.meduniwien.ac.at/ethik-kommission/mitglieder/ abrufbar. Mitglieder der Ethik-Kommission, die für diesen Tagesordnungspunkt als befangen anzusehen waren und daher laut Geschäftsordnung an der Entscheidungsfindung/Abstimmung nicht teilgenommen haben: Frau Ass.Prof.Dr. Elisabeth Schwendenwein

ACHTUNG: Unter Berücksichtigung der "ICH-Guideline for Good Clinical Practice" gilt dieser Beschluss **ein Jahr ab Datum der Ausstellung**. Gegebenenfalls hat der Antragsteller eine Verlängerung der Gültigkeit rechtzeitig zu beantragen.

Dieses Votum ist für berechtigte Benutzer/innen in digitaler Form unter der Adresse https://ekmeduniwien.at/vote/6029/download/ abrufbar.

**Signaturwert**	rWQD+YZLA7GtsKMVsvVYE+ZenB/fLVVCbFZPuOWVJkFXFaW9sujLe59Ns+pbSFcA4pK772Illdn3pc4+KH9Q9w==	
	**Unterzeichner**	Dr. Jürgen Zezula
	**Aussteller-Zertifikat**	CN=a-sign-Premium-Sig-02,OU=a-sign-Premium-Sig-02,O=A-Trust Ges. f. Sicherheitssysteme im elektr. Datenverkehr GmbH,C=AT
	**Serien-Nr.**	851965
	**Methode**	urn:pdfsigfilter:bka.gv.at:binaer:v1.1.0
	**Parameter**	etsi-moc-1.1@c4b61009
**Prüfinformation**	Informationen zur Prüfung der elektronischen Signatur und des Ausdrucks finden Sie unter: http://www.signaturpruefung.gv.at	
**Datum/Zeit-UTC**	2015-03-03T13:04:27Z	
