# Supplementary material for: Redifferentiation of aged human articular chondrocytes by combining bone morphogenetic protein-2 and melanoma inhibitory activity protein in 3D-culture
Source: PLoS One. 2017 Jul 13;12(7):e0179729. doi: 10.1371/journal.pone.0179729 (PMC5509113; doi:10.1371/journal.pone.0179729)
Supplement: S2 Fig — Minimal data set for each gene and the GAG/DNA. (PDF) [file pone.0179729.s002.pdf]

AGG	Pat 1	Pat 2	Pat 3	Pat 4
nativ	-4,91523997	-5,914409	-6,24706459	-6,00255712
cells	4,421349843	2,998886108	2,495827993	1,815649033
T0	4,288183212	2,432277679	3,662451426	4,486216227
KO T14	3,588055929	2,620170593	5,634554545	2,651386897
BMP-2 T14	1,733494441	0,559060415	4,037547429	1,720329364
MIA T14	3,837286631	2,999416351	5,282990774	2,287733078
BMP-2+MIA T1	1,838376363	0,420794805	3,330835342	1,198797226
KO T28	5,137477875	3,597955068	2,848491033	2,124559402
BMP-2 T28	2,857503891	1,008516947	-0,18479983	-1,2655309
MIA T28	4,720187505	3,226214091	1,431992849	1,336001714
BMP-2+MIA T2	2,881733576	0,914332072	-0,78672854	0,82873853

ALP	Pat 1	Pat 2	Pat 3	Pat 4
nativ	3,896263758	0,834603628	2,52790006	3,123159409
cells	0,891838074	-5,24186834	-2,91868591	0,660390218
T0	2,423814774	-3,13064194	-1,65488434	1,366486231
KO T14	4,331734339	0,633441289	4,072978973	3,866392136
BMP-2 T14	2,158167521	0,338300705	3,521109263	3,457204819
MIA T14	1,963727315	0,09557279	3,687173843	2,703243256
BMP-2+MIA T1	2,622378667	0,79609553	4,351796468	2,612984975
KO T28		2,220464706	1,061825434	4,094257355
BMP-2 T28	3,73372523	1,093444824	-0,25828616	0,903133392
MIA T28		1,793823878	0,38269647	1,865540822
BMP-2+MIA T2	3,878329595	1,21863238	-1,06714535	1,334344228

col10	Pat 1	Pat 2	Pat 3	Pat 4
nativ	-2,03934288	-2,30216789	-1,78689384	-5,1783371
cells	8,802717209	6,9596831	6,115561803	6,064132055
T0	6,097417196	5,339131037	5,51598231	3,418610891
KO T14	5,102690379	4,598126094		
BMP-2 T14	5,193215052	4,335260391		3,92424202
MIA T14	5,336533864	4,667580922	5,687301636	4,949061712
BMP-2+MIA T1	4,335866292	4,825403214	7,753721873	4,932552973
KO T28		6,802767436	6,449911753	4,237990697
BMP-2 T28	7,026217779	4,378742218	3,831216812	0,156376521
MIA T28		5,215432485	6,380176544	4,27036794
BMP-2+MIA T28		4,600464503	4,265830358	1,467092514

col1	Pat 1	Pat 2	Pat 3	Pat 4
nativ	7,202253342	7,62383906	9,11750857	8,639199575
cells	1,629346848	0,395573934	3,771579742	2,904432297
T0	3,058175405	1,751988729	5,753714879	4,942937851
KO T14	3,94022433	4,576066335	8,306263606	7,625164668
BMP-2 T14	3,750693639	4,378725688	7,837693532	7,048083623
MIA T14	3,937686284	4,876559575	7,962104162	7,077723185
BMP-2+MIA T1	3,807162603	4,346617381	7,436681112	7,271823883
KO T28	8,18103981	6,436776479	8,07261912	6,431283951
BMP-2 T28	5,739674886	4,326270421	7,528301875	4,586012522
MIA T28	7,198013941	6,163972855	6,868419011	6,050945282
BMP-2+MIA T2	5,808568319	4,021904627	6,92657725	6,134713491

col2	Pat 1	Pat 2	Pat 3	Pat 4

nativ	-1,29489072	-1,01905187	-1,72524897	0,06172053
cells	13,26780383	9,89264679	11,52918434	8,907390594
T0	11,04847145	7,382914861	10,10413043	11,72312101
KO T14	8,318354289	7,549781164	9,658455531	6,083105087
BMP-2 T14	2,942163467	1,706183116	6,856913249	3,399333954
MIA T14	8,314937592	7,724913279	9,575895309	6,121281306
BMP-2+MIA T1	3,230741501	1,380702337	6,343681971	3,997080485
KO T28	10,54615657	8,215925217	7,82192866	4,62999026
BMP-2 T28	4,388320287	0,937886556	0,147839228	-1,72646904
MIA T28	10,39599864	7,655617396	4,666345596	2,8167394
BMP-2+MIA T2	4,265539169	0,493754069	-0,80363655	0,995781581

GAG/DNA	T0	KO T14	BMP-2 T14	MIA T14
Pat1	0,082656162	0,140158949	0,235131584	0,25247457
Pat 2	0,117468606	0,223678704	0,162458471	0,107436844
Pat 3	0,13470676	0,257946021	0,215359361	0,1902796
Pat4	0,103419676	0,199431836	0,225030477	0,199959011

	BMP-2+MIA T14
Pat1	0,180349805
Pat 2	0,188091442
Pat 3	0,14614548
Pat4	0,179936635

Pat1	KO T28	BMP-2 T28	MIA T28	BMP-2+MIA T28
Pat 2	0,190496931	0,217050178	0,197913861	0,154932891
Pat 3	0,214628969	0,157032432	0,221356445	0,20432258
Pat4	0,232387015	0,109259101	0,203893804	0,134767624
	0,251902678	0,167130157	0,413842475	0,147071187
